# A New Strategy in Observer Modeling for Greenhouse Cucumber Seedling Growth

**DOI:** 10.3389/fpls.2017.01297

**Published:** 2017-08-08

**Authors:** Quan Qiu, Chenfei Zheng, Wenping Wang, Xiaojun Qiao, He Bai, Jingquan Yu, Kai Shi

**Affiliations:** ^1^Beijing Research Center of Intelligent Equipment for Agriculture, Beijing Academy of Agriculture and Forestry Sciences Beijing, China; ^2^Department of Horticulture, Zhejiang University Hangzhou, China; ^3^Department of Mechanical and Aerospace Engineering, Oklahoma State University Stillwater, OK, United States

**Keywords:** crop physiological information, state observer, greenhouse, cucumber seeding growth, canonical correlation analysis, support vector machine

## Abstract

State observer is an essential component in computerized control loops for greenhouse-crop systems. However, the current accomplishments of observer modeling for greenhouse-crop systems mainly focus on mass/energy balance, ignoring physiological responses of crops. As a result, state observers for crop physiological responses are rarely developed, and control operations are typically made based on experience rather than actual crop requirements. In addition, existing observer models require a large number of parameters, leading to heavy computational load and poor application feasibility. To address these problems, we present a new state observer modeling strategy that takes both environmental information and crop physiological responses into consideration during the observer modeling process. Using greenhouse cucumber seedlings as an instance, we sample 10 physiological parameters of cucumber seedlings at different time point during the exponential growth stage, and employ them to build growth state observers together with 8 environmental parameters. Support vector machine (SVM) acts as the mathematical tool for observer modeling. Canonical correlation analysis (CCA) is used to select the dominant environmental and physiological parameters in the modeling process. With the dominant parameters, simplified observer models are built and tested. We conduct contrast experiments with different input parameter combinations on simplified and un-simplified observers. Experimental results indicate that physiological information can improve the prediction accuracies of the growth state observers. Furthermore, the simplified observer models can give equivalent or even better performance than the un-simplified ones, which verifies the feasibility of CCA. The current study can enable state observers to reflect crop requirements and make them feasible for applications with simplified shapes, which is significant for developing intelligent greenhouse control systems for modern greenhouse production.

## Introduction

Greenhouse has been widely believed to be a powerful cultivation facility in large regions of the world. With its year-round running capability, greenhouse can greatly extend the productivity of farmland. Inner climate control is the key factor that endows greenhouse with the year-round running feature. It can serve the plants with optimal growth conditions while maximizing the grower's economic benefits. At the early stage, greenhouse climate control was executed based on growers' experience. As a result, the selection of control set-points and operation time suffered from heavy arbitrariness, which led to poor control performances and high energy cost.

To improve the control accuracy and reduce the energy cost, computerized greenhouse climate control based on sensing technologies was introduced. The existing computerized control strategies can be categorized into two branches: conventional control and generalized optimal control (Duarte-Galvan et al., 2012). Conventional control can be regarded as the early development stage of computerized control strategies. It only considers how to reduce the deviation between the set-points and the observations/measurements of interested values, such as inner temperature or humidity of greenhouse. Logic control (ON/OFF of actuators) strategies (Hooper and Davis, 1988) and proportional integral derivative (PID) control (Setiawan et al., 2000) are typical examples for conventional control. Compared with conventional control, generalized optimal control solves the climate control problem in a higher level by considering practical limitations, such as lack of suitable system model, actuator capabilities and energy consumption. Many optimal control strategies have been proposed, including predictive control (Roca et al., 2016), special optimal control (Van Beveren et al., 2015), adaptive control (Gerasimov and Lyzlova, 2014), neural networks control (Manonmani et al., 2016), fuzzy control (Azaza et al., 2015), nonlinear control (Zeng et al., 2012), robust control (Bennis et al., 2008), and multivariable control (Giraldo et al., 2016), etc. A comprehensive review of greenhouse control strategies is given by Van Straten et al. (2010).

All close-loop control systems need an essential component to report the states of the plant (control object), in order to decide when and how to take control actions. This component can be defined as “state observer.” In greenhouse control systems, state observer can be a sensor or a sensing data based model. Sensor observers usually appear in conventional control systems, and the states of the control objects can be directly obtained from sensor measurements, such as temperature and humidity. Model observers usually appear in optimal control systems, and the states of the control objects are generated by feeding sensor measurements into a model. Because greenhouse-crop system is a complex system, sensor observers using few parameters are usually not capable to obtain the true states of the system. As a result, model observers are drawing more and more attentions from researchers.

Models for greenhouse-crop systems are undergoing tremendous progress in last decades. A number of famous horticultural crop models were proposed, such as TOMGRO (Jones et al., 1991; Shamshiri et al., 2016), HORTISIM (Gijzen et al., 1998; Li et al., 2009), and TOMSIM (Heuvelink, 1999; Vaca et al., 2015). The popular horticultural crop models generally cover the similar topics with that of open field models, such as biomass production/yield modeling (Vanthoor et al., 2011; von Borell du Vernay, 2016), water relations modeling (Chen et al., 2014), plant nutrition modeling (Juárez-Maldonado et al., 2014), plant spatial structure and development modeling (Kang et al., 2011), influences of environmental control actuators (Pahuja et al., 2015), etc.

**Figure 1:**
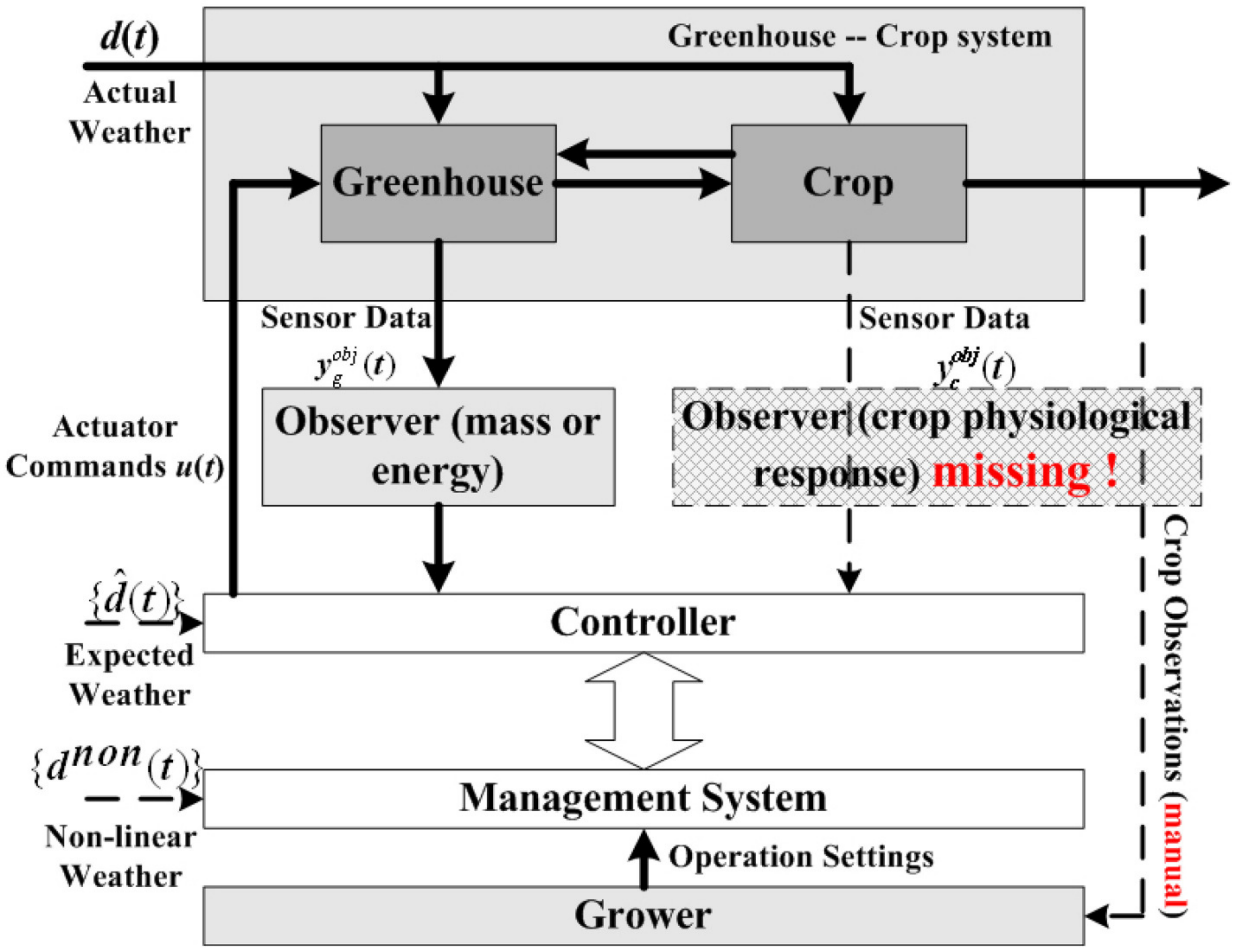
Typical control hierarchy for greenhouse-crop system (Van Straten et al., 2010). A typical greenhouse-crop system control loop consists of Greenhouse, Crop, Controller, Management System, and Grower. As the observer for crop responses is missing (the dash line rectangle), the Controller needs information from the Management System, whose operation settings are generated by Grower's manual observations.

Although much progress has been made, horticultural crop modeling still has a long way to go. One conspicuous drawback is the missing of observers for crop physiological response, which leads to the absence of true crop requirements in control decision making process. Figure 1 is a common control hierarchy of current greenhouse-crop systems, modified from the original version of Van Straten et al. (2010). As shown in Figure 1, observers for energy/mass transportation are employed to help controller make decisions, most existing crop models focus on this topic. However, information of crop growth states (shown with a dash line rectangle) is rarely used by the controller. Crop growth state observations (or predictions) are usually taken manually by experienced growers, rather than sensors or computerized observers. Another drawback of crop models is that the number of parameters in a model is typically large. For example, the TOMGRO model has 69 parameters for version 1.0 and 574 parameters for version 3.0 (Jones et al., 1999). A large number of parameters results in not only high computational load, but also poor model feasibility in applications (Speetjens et al., 2009).

Aiming at addressing the drawbacks identified above, we propose a new observer building strategy for greenhouse cucumber seedling growth. The encouraging studies on sensing technologies show that chlorophyll fluorescence (Maxwell and Johnson, 2000; Misra et al., 2012) has become biological probes to investigate the physical status of high plants (Bolhar-Nordenkampf et al., 1989; Ajigboye et al., 2016). Thus, it is expected that we can employ chlorophyll fluorescence and leaf gas exchanges parameters as a powerful tool to reveal plant growth status in response to changes of greenhouse environmental conditions (Nishina, 2015). On the other hand, the seedling nursery industry is booming with the specialization in horticultural production, as the quality of seedlings is vital for both vegetative growth and reproductive growth, such as the crop morphogenesis and flower bud differentiation. Even though the growth period of seedlings is short, mis-controls of greenhouse environment may result in large economic losses to growers (Moriyuki and Fukuda, 2016). Quick and precise control responses according to the inner requirements of seedlings are essential, and the research of crop physiological information embedded observer becomes crucial.

In the proposed observer, crop physiological information is measured by chlorophyll fluorescence technologies, and employed together with environmental parameters to predict growth status of cucumber seedling. Support Vector Machine (SVM) acts as the mathematical modeling tool for the observer. To simplify the model, Canonical Correlation Analysis (CCA) is used to find the dominant parameters. Experimental results demonstrate that physiological parameters can improve the prediction accuracies of growth state observers, and that simplified observer models using dominant parameter sets as the inputs can give equivalent or even better performances than observer models using complete parameter sets as the inputs.

## Materials and methods

### Canonical correlation analysis

Canonical correlation analysis is a branch of multivariate statistical analysis, which is good at handling correlation analysis for two sets of variants (Hardoon et al., 2004). Different from regression analysis, CCA not only focuses on the correlations between a set of dependant variants and one single independent variant, but also considers the correlations among independent variants from the same set. Following the same idea as Principle Component Analysis (PCA), CCA abstracts principle components from the dependant variant set and the independent variant set, respectively, and maximizes the correlation between the two sets of principle components. Then the correlation of the two principle component sets can be employed to describe the linear correlation between the dependant variants set and the independent variants set. CCA has been widely used in many research fields, such as computer vision and medical science.

The main idea of CCA can be elaborated as follows. We assume **X** and **Y** are two sets of random variants with correlation

(1) $$\begin{array}{r} X=\left(x_{1},x_{2},\cdots ,x_{p}\right)\prime \end{array}$$

(2) $$\begin{array}{r} Y=\left(y_{1},y_{2},\cdots ,y_{q}\right)\prime \end{array}$$

**X** has *p* component variants and **Y** has *q* component variants. Without loss of generality, we assume *p* ≤ *q*. Then, we use two aggregate variables *U* and *V* to express **X** and **Y** in new linear combinations as

(3) $$\begin{array}{r} U=a_{1}x_{1}+a_{2}x_{2}+\cdot \cdot \cdot a_{p}x_{p}\equiv {\text{a}}^{\prime }\text{X} \end{array}$$

(4) $$\begin{array}{r} V=b_{1}y_{1}+b_{2}y_{2}+\cdot \cdot \cdot +b_{q}y_{q}\equiv {\text{b}}^{\prime }\text{Y} \end{array}$$

where *U* and *V* are a pair of Canonical Correlation Variants (CCV) of **X** and **Y**, **a** = [*a*_1_, *a*_2_, ⋯, *a*_*p*_] and **b** = [*b*_1_, *b*_2_, ⋯, *b*_*q*_] are the coefficients of CCV, Theoretically, there are numerous pairs of **a** and **b**. We need to find the pairs showing maximum correlations of **X** and **Y**, which is equivalent to maximize the covariance of *U* and *V*. Using the idea from PCA, we can define

(5) $$\begin{array}{r} \lambda^{2}=\max\left(\mathrm{cov}\left(U,V\right)\right) \end{array}$$

where λ is the Canonical Correlation Coefficient (CCC) for *U* and *V*. As the eigenvalue of Equation (5), λ can have *p* different values. Without loss of generality, we assume λ_1_ ≥ λ_2_ ≥ ⋯ ≥ λ_*p*_. And every λ_*i*_(*i* = 1, ⋯ , *p*) will determine a corresponding pair of coefficient sets ${\tilde{\text{a}}}_{i}$ and ${\tilde{\text{b}}}_{i}$. If we also assume var(*U*_*i*_) = var(*V*_*i*_) = 1 for computation convenience, ${\tilde{\text{a}}}_{i}$ and ${\tilde{\text{b}}}_{i}$ can be called as a pair of Standard Coefficient (SC) sets for λ_*i*_.

Besides the CCC and SC, CCA also has several key values in the analyzing process, including Canonical Loading, explanation proportion of CCV, and significance testing value of CCV.

Canonical Loading (CL) is also called structure of CCV. CL for **X** and *U* can be obtained as

(6) $$\begin{array}{r} \text{C}{\text{L}}_{\text{X}U}=\mathrm{cov}\left(\text{X},\text{U}\right) \end{array}$$

Similarly, CL for **Y** and *V* can be obtained as

(7) $$\begin{array}{r} \text{C}{\text{L}}_{\text{Y}V}=\mathrm{cov}\left(\text{Y},\text{V}\right) \end{array}$$

Explanation proportion for **X** and *U*_*i*_ is

(8) $$\begin{array}{r} m_{U_{i}}=\overset{p}{\underset{j=1}{\sum }}CL_{\text{X}U}{\left(j,i\right)}^{2}/p \end{array}$$

Explanation proportion for **Y** and *V*_*i*_ is

(9) $$\begin{array}{r} n_{V_{i}}=\overset{q}{\underset{j=1}{\sum }}CL_{\text{Y}V}{\left(j,i\right)}^{2}/q \end{array}$$

Significance testing value of CCV can be computed as

(10) $$\begin{array}{r} Q_{i}=-\left(l-i-\frac{p+q+1}{2}\right)\ln\;\left(\overset{p}{\underset{i}{\prod }}\left(1-\lambda_{i}^{2}\right)\right) \end{array}$$

When *l* is big enough, which means *l* > (*p* + *q* + 1)/2 + *k* (*k* is the number of the nonzero eigenvalues, usually *k* = *p*), we can infer that *Q*_*i*_ is approximate χ^2^[(*p*−*i*+1)(*q*−*i*+1)] distribution. As a result, under the testing lever α (usually α = 0.05), if

(11) $$\begin{array}{r} Q_{\text{i}}>\chi_{\alpha }^{2}\left[\left(p-i+1\right)\left(q-i+1\right)\right] \end{array}$$

we conclude that the *i*-th pair CCV is significance and should be used for CCA.

### Support vector machine

For growth modeling problems, the sample quantity is always small compared to the whole crop group. And the correlation complexity among environmental, physiological and growth parameters is far beyond the capacity of ordinary linear prediction tools. Support Vector Machine (Boser et al., 1992; Cortes and Vapnik, 1995) is a popular machine learning tool for classification and prediction. Compared with other tools, it has distinguishing advantages on handling small sample size problems, nonlinear classification/prediction problems and high dimensional classification/prediction problems. As a result, we choose SVM as the main modeling tool.

Kernel function is a key factor in SVM training. There are different kernel functions. Each kernel function has its unique characteristics and is good at handling a specific training set. There are 4 widely used kernel functions:

Linear:

(12) $$\begin{array}{r} K\left({\text{x}}_{i},{\text{x}}_{j}\right)={{\text{x}}_{i}}^{\text{T}}{\text{x}}_{j} \end{array}$$

Polynomial:

(13) $$\begin{array}{r} K\left({\text{x}}_{i},{\text{x}}_{j}\right)={\left(\gamma {{\text{x}}_{i}}^{\text{T}}{\text{x}}_{j}+r\right)}^{d},\gamma >0 \end{array}$$

Radial Basis Function (RBF):

(14) $$\begin{array}{r} K\left({\text{x}}_{i},{\text{x}}_{j}\right)=\exp\left(-\gamma {\left||{\text{x}}_{i}-{\text{x}}_{j}|\right|}^{\text{2}}\right),\gamma >0 \end{array}$$

Sigmoid:

(15) $$\begin{array}{r} K\left({\text{x}}_{i},{\text{x}}_{j}\right)=\tanh\left(\gamma {{\text{x}}_{i}}^{\text{T}}{\text{x}}_{j}+r\right) \end{array}$$

Here, γ, *r*, and *d* are parameters of kernel functions. According to A Practical Guide to Support Vector Classification (Hsu et al., 2003), RBF shows superior performances in computation complexity and exceptional situation handling. The linear kernel can be considered as a special case of RBF. Sigmoid kernel has similar behavior to RBF under certain parameter settings. That is, RBF can cover most cases of linear and sigmoid kernels. Compared with polynomial kernel, RBF employs fewer hyper parameters and does all the computation within the region of [0, 1], resulting in a greatly reduced training and testing computation load. In this paper, Linear kernel and RBF kernel are used for prediction.

### Data acquisition

#### Plant material and growth conditions

Seeds of cucumber genotypes “JINYOU NO.4” were germinated and grown in a medium containing a mixture of peat, vermiculite and perlite (6:3:1) in plastic pots (diameter, 10.5 cm; depth, 17.5 cm) in a controlled environment. One seedling was grown per pot. The growth conditions were as follows: the photosynthetic photo flux density (PPFD) was 400 μmol•m^−2^•s^−1^, the photoperiod was 14/10 h (day/night), the day/night air temperature was 26/22°C and the relative humidity was 75%. Seedlings were watered daily to maintain optimum moisture and were fertilized with Hoagland's nutrient solution every 3 days. About 2 weeks after germination, the seedlings at 2-true leaves stage were transferred to different controlled-environment growth room, where the atmospheric environment including average temperature during the daytime (T_D, °C), average temperature during the night (T_N, °C), carbon dioxide concentration (CO_2_, μmolCO_2_•mol^−1^), relative humidity (RH, %), absolute humidity (AH, μmolH_2_O•mol^−1^), light intensity (PARo, μmol•m^−2^•s^−1^), ratio of white light and blue light (Ratio_W/B), ration of white light and red light (Ratio_W/R) were controlled at different but stable levels. For all cases, unless otherwise stated, root substrate management such as water, nutrient supply and others were the same, which are not considered as environmental variants in the current experiments. To get reliable growth responses, we designed 13 different environmental parameter combinations (Table 1). In each combination, at least one environmental parameter is different from other combinations. As a result, we had 13 sub-experiments, and each sub-experiment runs 9 days. On days 0, 3, 6, and 9 after different environment treatment, at least 5 biological replicates were taken from each grown-condition for growth rate determination. For physiological status parameters, i.e., leaf gas exchange and chlorophyll fluorescence, there are also 2 repeat measurements for each 5–8 biological replicates. When the data collection process was finished, we got 73 cucumber seedling samples. Each sample consisted of 1 set of environmental data (8 parameters), 4 sets of physiological data (10 parameters on days 0, 3, 6, 9, respectively), and 3 sets of growth state data (4 parameters on days 3, 6, 9, respectively).

**Table 1:** Thirteen different environmental parameter combinations.

**Trials**	**T_D**	**T_N**	**CO_2_**	**RH**	**AH**	**PARo**	**Ratio_W/B**	**Ratio_W/R**
1	25	20	800	26	72	130	0.96: 1	0.96: 9.52
2	25	21	400	17	46	100	1.52: 1	1.52: 1.23
3	27	22	400	18	45	115	1.52: 1	1.52: 1.23
4	25	21	400	20	55	175	0.06: 1	0.06: 8.04
5	25	21	400	21	52	162	0.96: 1	0.96: 9.52
6	25	21	400	21	46	188	2.47: 1	2.47: 8.78
7	24	20	400	16	47	416	0.03: 2.76	0.03: 1
8	26	22	400	14	40	666	0.01: 1.3	0.01: 1
9	26	22	400	26	60	184	0.01: 1.54	0.01: 1
10	25	25	400	21	54	40	1.52: 1	1.52: 1.23
11	18	18	400	16	55	30	1.52: 1	1.52: 1.23
12	25	21	400	25	74	53	0.96: 1	0.96: 9.52
13	35	25	450	18	31	150	0.96: 1	0.96: 9.52

*In each combination, at least one environmental parameter was different from other combinations. As a result, we had 13 sub-experiments, and each sub-experiment runs 9 days. All the environmental parameters were kept stable in the sub-experiment*.

#### Growth measurement

Cucumber plants from each grown-condition were sampled randomly for determination of average plant height increment (Plant_Height, cm•d^−1^), average leaf area increment (Leaf_Area, cm^2^•d^−1^), average fresh weight increment (Fresh_Weight, g•d^−1^), average dry weight increment (Dry_Weight, g•d^−1^). Total leaf area per plant was determined by measuring the length and width of each leaf and calculating leaf area using the equation of Cho et al. (2007). After fresh weight was determined, plants were dried to constant dry mass in an oven at 80°C. The average plant growth rate was calculated on the basis of per day. Plant_Height and Leaf_Area were measured on the day of 0, 3, 6, and 9. Fresh_Weight and Dry_Weight were measured only on the day of 9. Finally, we have 3 sets of growth state parameters for each sample as G1, G2, and G3.

#### Physiological parameter measurement

Leaf gas exchange and chlorophyll fluorescence analysis were conducted to measure physiological parameters. Leaf gas exchange measurements were coupled with measurements of chlorophyll fluorescence using an open gas exchange system (LI-6400; LI-COR, Inc., Lincoln, NE, USA) with an integrated fluorescence chamber head (LI-6400-40 leaf chamber fluorometer; LI-COR, Inc.) on the second fully developed leaves in the morning from 9:00 to 11:00. For all cases, during gas exchange and chlorophyll fluorescence parameters analysis, the environment condition, such as temperature, relative humidity, CO_2_ concentration were kept as the same environment where the seedlings grew, by putting the Inlet-connected buffer gas cylinder in the same growth chamber, while the incident PPFD were set as the same value that the built-in light sensor sensed. The main leaf gas exchange and chlorophyll fluorescence parameters including net photosynthesis rate (Pn, μmolCO_2_• m^−2^•s^−1^), stomatal conductance (Cond, molH_2_O• m^−2^•s^−1^), intercellular CO_2_ concentration (Ci, μmolCO_2_•mol^−1^), efficiency of excitation capture by open PSII center (Fv′/Fm′, [0, 1]), quantum efficiency of PSII (PhiPS2, [0, 1]), quantum efficiency of CO_2_ fixation (PhiCO_2_, [0, 1]), photochemical quenching coefficient (qP, [0, 1]), electron transport rate (ETR, μmolCO_2_• m^−2^•s^−1^), transpiration rate (Tr, molH_2_O•m^−2^•s^−1^), and vapor pressure deficit at the leaf temperature (VpdL, kPa) were taken. Fluorescence parameters were calculated on the basis of the light-adapted fluorescence measurements. The PhiPS2 = (*F*′_*m*_-*F*_*s*_)/*F*′_*m*_, F′_*v*_/F′_*m*_ = (*F*′_*m*_-*F*′_0_)/*F*′_*m*_, qP = (*F*′_*m*_-*F*_*s*_)/(*F*′_*m*_-*F*′_0_) (Genty et al., 1989; Van Kooten and Snel, 1990). All physiological parameters were measured on the day of 0, 3, 6, and 9. Thus, we have 4 sets of physiological parameters for each sample as P1, P2, P3, and P4.

## Results

### Results of canonical correlation analysis

The aim of CCA is to find inconsequential environmental/physiological parameters for growth observation. Since the inconsequential parameters do not appear in the observer model, the model will be simplified and more suitable for control applications. In this section, we analyzed the parameter correlations in terms of groups: (environmental, growth) and (physiological, growth). As mentioned above, we have chosen 8 environmental parameters, 10 physiological parameters and 4 growth parameters. All the CCA results for (E, G) and (P, G) groups are given in the Supplementary Material.

#### CCA for environmental and growth parameters

In this part, we analyze the correlations between 1 environmental data set and 3 growth state data sets. CCA is performed for 3 groups of parameters: (E, G1), (E, G2), and (E, G3). The CCA results reveal the influences of different environmental parameters on different cucumber seedling growth period: early stage (0 ~ 3 days), middle stage (4 ~ 6 days), and late stage (7 ~ 9 days). Without loss of generality, we denote T_D by e1, T_N by e2, CO_2_ by e3, RH by e4, AH by e5, PARo by e6, Ratio_W/B by e7, Ratio_W/R by e8, Plant_Height by g1, Leaf_Area by g2, Fresh_Weight by g3, and Dry_Weight by g4. Thus, for the CCA of each (environmental, growth) group, we have *p* = 8, *q* = 4, and min(*p, q*) = 4. It follows that there are 4 pairs of CCV for each group. For example, the key values of CCV for the (E, G1) group are listed in Tables 2–**5**.

**Table 2:** Canonical correlation coefficients of (E, G1).

**λ_1_**	**λ_2_**	**λ_3_**	**λ_4_**
0.918148381	0.845727042	0.619930676	0.450549586

To analyze the relationship between two parameter sets, we employ Correlation Loading (CL) as the main correlation analyzing tool. For (E, G1), we have 4 sets of CL. Significant testing and explanation proportion allow us to determine which sets should be taken into consideration. Referring to the significance testing results (Table 3), we have $Q_{\text{i}}>\chi_{\alpha }^{2}\left[\left(p-i+1\right)\left(q-i+1\right)\right]$ for λ_1_, λ_2_, λ_3_, and λ_4_ under the testing level of α = 0.05. Based on these results, we conclude that all the 4 pairs of CCV are significant. We further consider the explanation proportion (shown in Table 4). For the first and second pairs of CCV, the explanation proportions of *U*_*i*_ for E and *V*_*i*_ for G1, are all above 0.1 and show a nearly balanced state in values. For the third pair, the explanation proportion of *U*_*i*_ for E is smaller than 0.05, while the proportion of *V*_*i*_ for G1 is more than 0.39. Because the proportions show an unbalanced state and one of them is too small to be meaningful, the third pair of CCV is not considered in our CCA for (E, G1). We also drop the fourth pair because the significant testing value is not prominent enough. As a result, we only use the first two sets of CL to conduct the CCA of (E, G1). We call the CL used in CCA as CCA concerned CL.

**Table 3:** Significance testing of (E, G1).

	**λ_1_**	**λ_2_**	**λ_3_**	**λ_4_**
*Q*_*i*_	388.9466864	221.5749247	99.45613576	37.4272967
$\chi_{\alpha =0.05}^{2}\left[\left(p-i+1\right)\left(q-i+1\right)\right]$	46.194	32.671	21.026	11.070
(*p*−*i* + 1)(*q*−*i* + 1)	32	21	12	5

*Forλ_i_, if Q_i_ > χ^2^, λ_i_ is significant and the corresponding canonical correlation variants pair (U_i_, V_i_) is considered in the Canonical Correlation Analysis with a high probability. Here, every λ_i_ is significant*.

**Table 4:** Explanation proportion of (E, G1).

	**Explanation proportion** ***U*** **for E**				
	***U*_1_**	***U*_2_**	***U*_3_**	***U*_4_**	***Sum***
E	0.165105925	0.12477866	0.044655352	0.212473005	0.547012942
	**Explanation proportion V for G1**				
	***V***_1_	***V***_2_	***V***_3_	***V***_4_	***Sum***
G1	0.242347816	0.164395701	0.392923172	0.20033331	1

*Explanation Proportion (EP) helps Significance Testing select CCV pairs (U_i_, V_i_) for CCA. If the EP values of the same pair of (U_i_, V_i_) are balanced and large enough, and the corresponding λ_i_ is significant, then (U_i_, V_i_) is considered in CCA. “Balanced” means the difference of the two EP values is not large. For example, the two EP values of pair (U_1_, V_1_) are 0.165105925 and 0.242347816, respectively, and they are balanced; the two EP values of pair (U_3_, V_3_) are 0.044655352 and 0.392923172, respectively, and they are not balanced*.

From the CCA concerned CL of (E, G1) shown in Table 5, we can see that *U*_1_ correlates with e2, e3, e4, e5 (the absolute values are larger than 0.3), especially with e3 (the absolute value is larger than 0.5); *U*_2_ correlates with e2, e4, e6, e7, e8, especially with e2; *V*_1_ only correlates with g1; *V*_2_ correlates with g1, g2, g3, especially g3. In summary, the 7 environmental parameters including e2, e3, e4, e5, e6, e7, e8 have influences on g1, g2, g3 through CCV.

**Table 5:** Correlation loading of (E, G1).

	**Correlation loading for** ***U*** **and E**			
	***U*_1_**	***U*_2_**	***U*_3_**	***U*_4_**
*a*_1_	−0.013078091	0.099097725	−0.340607888	0.250697361
*a*_2_	0.330681294	0.584721841	−0.234375711	0.199345914
*a*_3_	−0.915494942	0.250538624	0.25421471	0.010319347
*a*_4_	−0.377940741	0.400936862	−0.079642597	−0.189197335
*a*_5_	−0.433440866	0.19730076	0.216262731	−0.424967005
*a*_6_	−0.016769029	−0.380659435	0.032961724	0.776785745
*a*_7_	0.026914194	0.306926582	0.044116542	−0.682815388
*a*_8_	0.203665157	0.380731142	−0.255981896	−0.557731164
	**Correlation loading for** ***V*** **and G1**			
	***V***_1_	***V***_2_	***V***_3_	***V***_4_
*b*_1_	0.9413701	0.33646808	0.006374862	−0.02389409
*b*_2_	0.152711533	0.31826233	0.396451757	−0.847475239
*b*_3_	0.031355557	0.646045495	0.71653988	−0.261175511
*b*_4_	0.242713034	0.160331978	0.949235827	0.119730469

*Correlation Loading (CL) helps to determine whether a parameter of one set has correlation with the other parameter set. For a parameter, if at least one CL's absolute value is above 0.3, and the corresponding CCV is considered in CCA, we consider that the parameter has correlation with the other parameter set. For example, the CL of a_2_ for U_1_ is −0.915494942, and (U_1_, V_1_) is considered in CCA, we consider that e2 (T_N) has correlation with growth parameters in G1. At the same time, even though the CL of a_1_ for U_3_ is −0.340607888, we do not consider that e1 (T_D) has correlation with growth parameters in G1, because (U_3_, V_3_) is not considered in CCA*.

Following the same analysis steps, we also run CCA for the groups of (E, G2) and (E, G3). Based on the analyze results, we find that the correlations of the two groups are similar to each other: e1, e2, e4, e6, e7, e8 have influences on g1, g2, g3, g4 through CCV.

**Figure 2:**
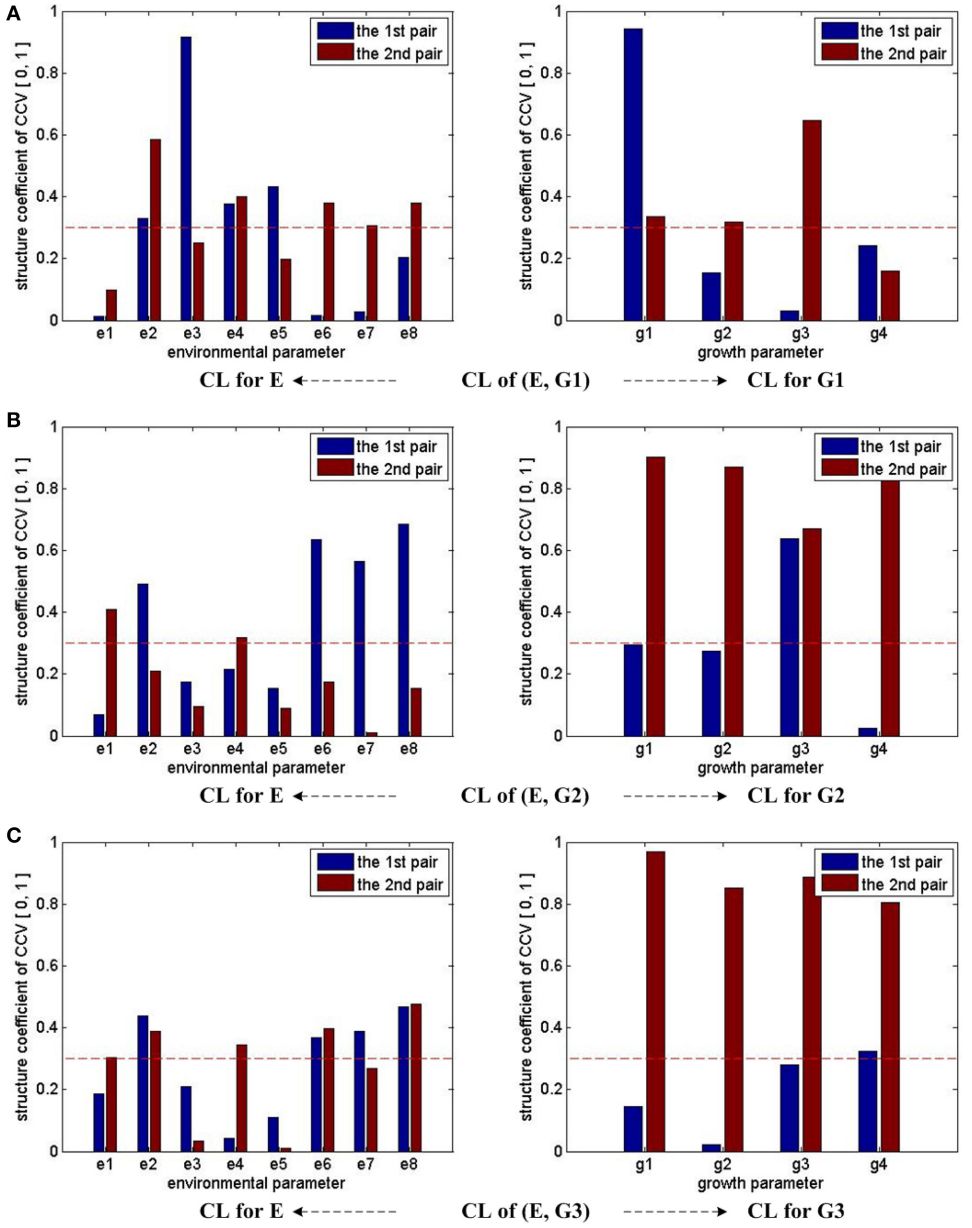
CCA concerned CL bars for (E, G1), (E, G2), and (E, G3). For (E, G1), (E, G2), and (E, G3), as only the first two pairs of CCV are concerned by CCA, there are two bars for each parameter in all the sub-figures. For each parameter, if one bar reaches the red dash line, we consider that the corresponding parameter has correlation with the other parameter set in the CCA. Taking the sub-figure **(A)** CL of group (E, G1)—CL for E (the sub-figure on the left of the first row) for example, none of e1's bar reaches the dash line. Thus, we conclude the environmental parameter e1 (T_D) has no/weak correlation with growth parameters in G1.

Figure 2 summarizes the CCA results for different (E, G) groups mentioned above. The height of each bar stands for the absolute value of the corresponding CL coefficient. Since the first two pairs of CCV are considered for each (E, G) pair, there are two bars for each environmental or growth parameter. We use 0.3 as the threshold value of the CL coefficients to determine whether the correlations are remarkable or not. In Figure 2, the threshold is marked with a red dash line. If any bar of a parameter reaches the red dash line, we consider that it has remarkable correlations with parameters in the other set. Following this rule, we conclude that e2, e4, e6, e7, e8 have strong influences on growth parameters during the whole experiment period, e1's influences on growth parameters are weak during the early stage (0 ~ 3 days), and e3 and e5's influences on growth parameters are weak during the middle (4 ~ 6 days) and late (7 ~ 9 days) seedling stages.

Based on the CCA results of three (E, G) groups, we conclude that e3 and e5 have weak influences on growth parameters during the middle and late seedling stages. As a result, a simplified environmental parameter combination of 6 dimensions is given by

(16) $$\begin{array}{r} \text{E}\_6\text{D}=\left[e1,\;e2,\;e4,\;e6,\;e7,\;e8\right] \end{array}$$

compared to the original environmental parameter combination of 8 dimensions

(17) $$\begin{array}{r} \text{E}\_8\text{D}=\left[e1,\;e2,\;e3,\;e4,\;e5,\;e6,\;e7,\;e8\right] \end{array}$$

Both E_6D and E_8D will be used in the observer building process for the purpose of comparison. If E_6D can deliver similar observation performances to E_8D, a simplified observer can be built based on E_6D.

#### CCA for physiological and growth parameters

In this part, we analyze the correlations between 4 physiological data sets and 3 growth state data sets. CCA is performed for 6 groups: (P2, G1), (P3, G2), (P4, G3) and (P1, G3), (P2, G3), (P3, G3). The CCA results of the first 3 groups reveal the correlations between physiological parameters and growth parameters belonging to the same stage: (P2, G1) for early stage (0 ~ 3 days), (P3, G2) for middle stage (4 ~ 6 days), and (P4, G3) for late stage (7 ~ 9 days). The CCA results of the last 3 groups reveal the correlations between physiological parameters of different stages and the final growth state parameters. Without loss of generality, we denote Pn by p1, Cond by p2, Ci by p3, Fv′/Fm′ by p4, PhiPS2 by p5, PhiCO_2_ by p6, qP by p7, ETR by p8, Tr by p9, VpdL by p10, Plant_Height by g1, Leaf_Area by g2, Fresh_Weight by g3, and Dry_Weight by g4. Thus, for the CCA of each (physiological, growth) pair, we have *p* = 10, *q* = 4, and min(*p, q*) = 4. Thus, there are 4 pairs of CCV. For example, the key values of CCV for the (P2, G1) group are listed in Tables 6–**9**.

**Table 6:** Canonical correlation coefficients of (P2, G1).

**λ_1_**	**λ_2_**	**λ_3_**	**λ_4_**
0.934281189	0.774302253	0.685212927	0.539731453

For (P2, G1), we have 4 sets of CL. Referring to the significance testing results (Table 7), we have $Q_{\text{i}}>\chi_{\alpha }^{2}\left[\left(p-i+1\right)\left(q-i+1\right)\right]$ for λ_1_, λ_2_, λ_3_, and λ_4_ under the testing level of α = 0.05. Based on these results, we conclude that all the 4 pairs of CCV are significant. We further consider the explanation proportion (shown in Table 8). For the first, second and third pairs of CCV, the explanation proportions of *U*_*i*_ for **P**2 and *V*_*i*_ for G1 are all above 0.1 and show a nearly balanced state in values. For the fourth pair, the explanation proportion of *U*_*i*_ for **P**2 is smaller than 0.04, while the proportion of *V*_*i*_ for G1 is more than 0.33. Because the proportions show an unbalanced state and one of them is too small to be meaningful, the fourth pair of CCV is not considered in our CCA for (P2, G1). As a result, we only use the first three pairs of CCV to do the CCA of (P2, G1).

**Table 7:** Significance testing of (P2, G1).

	**λ_1_**	**λ_2_**	**λ_3_**	**λ_4_**
*Q*_*i*_	255.1677841	120.2344164	61.14400305	21.17673426
$\chi_{\alpha =0.05}^{2}\left[\left(p-i+1\right)\left[\right]\left(q-i+1\right)\right]$	55.76	40.11	26.3	14.07
(*p* −*i* + 1)(*q* −*i* + 1)	40	27	16	7

*For λ_i_, if Q_i_ > χ^2^, λ_i_ is significant and the corresponding Canonical Correlation Variants pair (U_i_, V_i_) is considered in the Canonical Correlation Analysis with a high probability. Here, every λ_i_ is significant*.

**Table 8:** Explanation proportion of (P2, G1).

	**Explanation proportion** ***U*** **for P2**				
	***U*_1_**	***U*_2_**	***U*_3_**	***U*_4_**	***Sum***
P2	0.251407114	0.236510084	0.157155614	0.036876574	0.681949385
	**Explanation proportion V for G1**				
	***V***_1_	***V***_2_	***V***_3_	***V***_4_	***Sum***
G1	0.327508541	0.20870704	0.130351894	0.333432525	1

*The two EP values of pair (U_1_, V_1_) are 0.251407114 and 0.327508541, respectively, they are balanced; the two EP values of pair (U_4_, V_4_) are 0.036876574 and 0.333432525, respectively, they are not balanced. The contributions of U_4_ in explaining the correlation between P2 and G1 are too small to be considered. So the CCV pair (U_4_, V_4_) will not be used for CCA, even though λ_4_ is significant. As a result, we concern 3 pairs of CCV for CCA here, including (U_1_, V_1_), (U_2_, V_2_), and (U_3_, V_3_)*.

From the CCA concerned CL of (P2, G1) shown in Table 9, we can see that *U*_1_ correlates with p1, p3, p5, p6, p7, p8, p10 (the absolute values are larger than 0.3), especially with p1, p3 and p6 (the absolute values are larger than 0.5); *U*_2_ correlates with p1, p2, p4, p5, p6, p7, p8, p9, especially with p1, p2, p4, p6, p9; *U*_3_ correlates with p4, p5, p7, p8; *V*_1_ correlates with g1, g2, g4, especially with g1; *V*_2_ correlates with g3, g4, especially with g4; *V*_3_ correlates with g3. In summary, all the 10 physiological parameters have influences on 4 growth parameters through CCV.

**Table 9:** Correlation loading of (P2, G1).

	**Correlation loading for** ***U*** **and P2**			
	***U*_1_**	***U*_2_**	***U*_3_**	***U*_4_**
*a*_1_	−0.642981561	−0.602100812	0.219207031	0.097569221
*a*_2_	−0.202257745	−0.576443819	0.165279283	−0.207058935
*a*_3_	−0.930591559	−0.146724763	−0.123346051	−0.174737214
*a*_4_	−0.244819091	−0.528424665	−0.474203996	−0.234480666
*a*_5_	−0.456594265	−0.457973704	0.590766857	0.220701362
*a*_6_	−0.643725407	−0.600863709	0.218393613	0.097633487
*a*_7_	−0.377600166	−0.329525566	0.641263404	0.290897154
*a*_8_	−0.455518198	−0.4591943	0.591726819	0.221130685
*a*_9_	−0.02931568	−0.683987972	0.219437546	−0.197623722
*a*_10_	0.400002216	−0.107070231	0.223360753	−0.006469139
	**Correlation loading for** ***V*** **and G1**			
	***V***_1_	***V***_2_	***V***_3_	***V***_4_
*b*_1_	0.986642955	0.15157808	−0.050539181	−0.031710519
*b*_2_	0.323177915	−0.207551591	0.670767992	−0.634467236
*b*_3_	0.289325947	−0.374448435	−0.021143823	−0.880699611
*b*_4_	0.38524846	−0.792819622	−0.261680352	−0.393120929

*Correlation Loading (CL) helps to determine whether a parameter of one set has correlation with the other parameter set. For a parameter, if at least one CL's absolute value is above 0.3, and the corresponding CCV is considered in CCA, we consider that the parameter has correlation with the other parameter set. For example, the CL of a_2_ for U_2_ is −0.576443819, and (U_2_, V_2_) is considered in CCA, we consider that p2 (Cond) has correlation with growth parameters in G1*.

Following the same analysis steps, we also run CCA for the groups of (P3, G2), (P4, G3), (P1, G3), (P2, G3), and (P3, G3).

**Figure 3:**
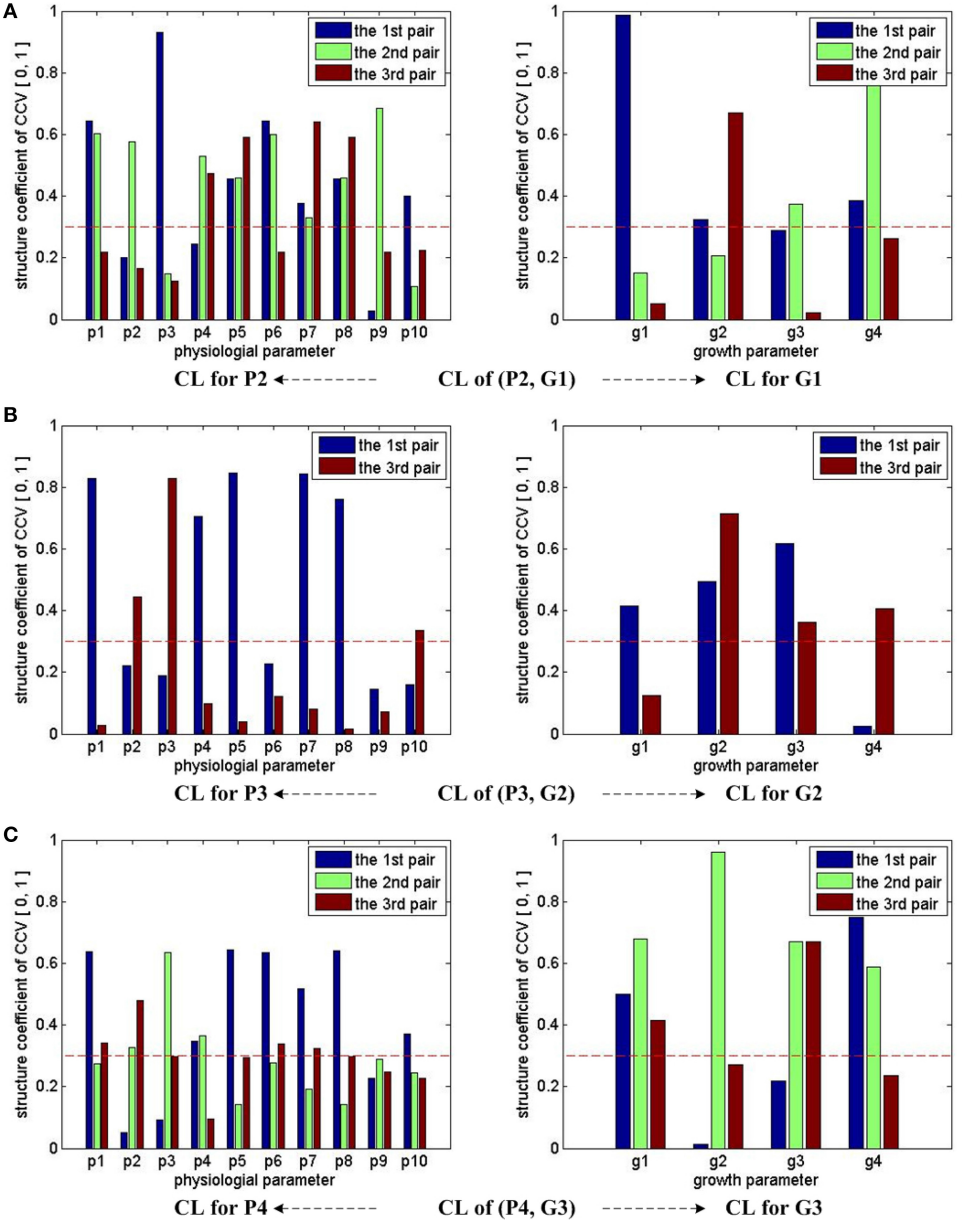
CCA concerned CL bars for (P2, G1), (P3, G2), and (P4, G3). For (P2, G1) and (P4, G3), the first three pairs of CCV are concerned by CCA, so we have 3 bars for each parameter in the first and third rows of sub-figures. For (P3, G2), two pairs of CCV are concerned by CCA, so we have 2 bars for each parameter in the second row of sub-figures. Taking the sub-figure **(B)** CL of group (P3, G2)—CL for P3 (the sub-figure on the left of the second row) for example, none of p9's bar reaches the dash line, we conclude that the physiological parameter p9 (Tr) in P3 has no/weak correlation with growth parameters in G2. Following the same rule, we can infer that p9 in P4 also has no/weak correlation with growth parameters in G3. All growth parameters in G1, G2, and G3 have correlation with physiological parameters.

Figure 3 summarizes the CCA results of groups (P2, G1), (P3, G2), and (P4, G3). Note that we consider the first three pairs of CCV for almost all the three groups, except (P3, G2), for which only the first and third pairs of its CCV are meaningful. With the help of the marked threshold line (red dash line), we conclude: for the group of (P2, G1), all physiological parameters have influences on growth parameters; for the group of (P3, G2), almost all physiological parameters have influences on growth parameters, except p6 and p9; for the group of (P4, G3), almost all physiological parameters have influences on growth parameters, except p9. In summary, we conclude that all 10 physiological parameters have strong influences on growth parameters during the early stage (0 ~ 3 days), p6 and p9's influences on middle stage (3 ~ 6 days) growth parameters are weak, and p9's influences on final stage (7 ~ 9 days) growth parameters are weak.

Based on the CCA results of (P3, G2), we conclude that p6 and p9 have weak influences on growth parameters during the middle seedling stages. As a result, a simplified physiological parameter combination of 8 dimensions is given by

(18) $$\begin{array}{r} \text{P}\_8\text{D}\_\text{6}\_\text{9}=\left[p1,\;p2,\;p3,\;p4,\;p5,\;p7,\;p8,\;p10\right] \end{array}$$

compared to the original physiological parameter combination

(19) $$\begin{array}{r} \text{P}\_10\text{D}=\left[p1,\;p2,\;p3,\;p4,\;p5,\;p6,\;p7,\;p8,\;p9,\;p10\right] \end{array}$$

P_8D_6_9 can be used for predicting G2. If P_8D_6_9 delivers similar performances as P_10D, a simplified observer can be built with P_8D_6_9.

Furthermore, based on the CCA results of (P4, G3), another simplified physiological parameter combination of 9 dimensions is given by

(20) $$\begin{array}{r} \text{P}\_9\text{D}\_\text{9}=\left[p1,\;p2,\;p3,\;p4,\;p5,\;p6,\;p7,\;p8,\;p10\right] \end{array}$$

P_9D_9 can be used for predicting G3. If P_9D_9 delivers similar performances as P_10D, a simplified observer can be built with P_9D_9.

**Figure 4:**
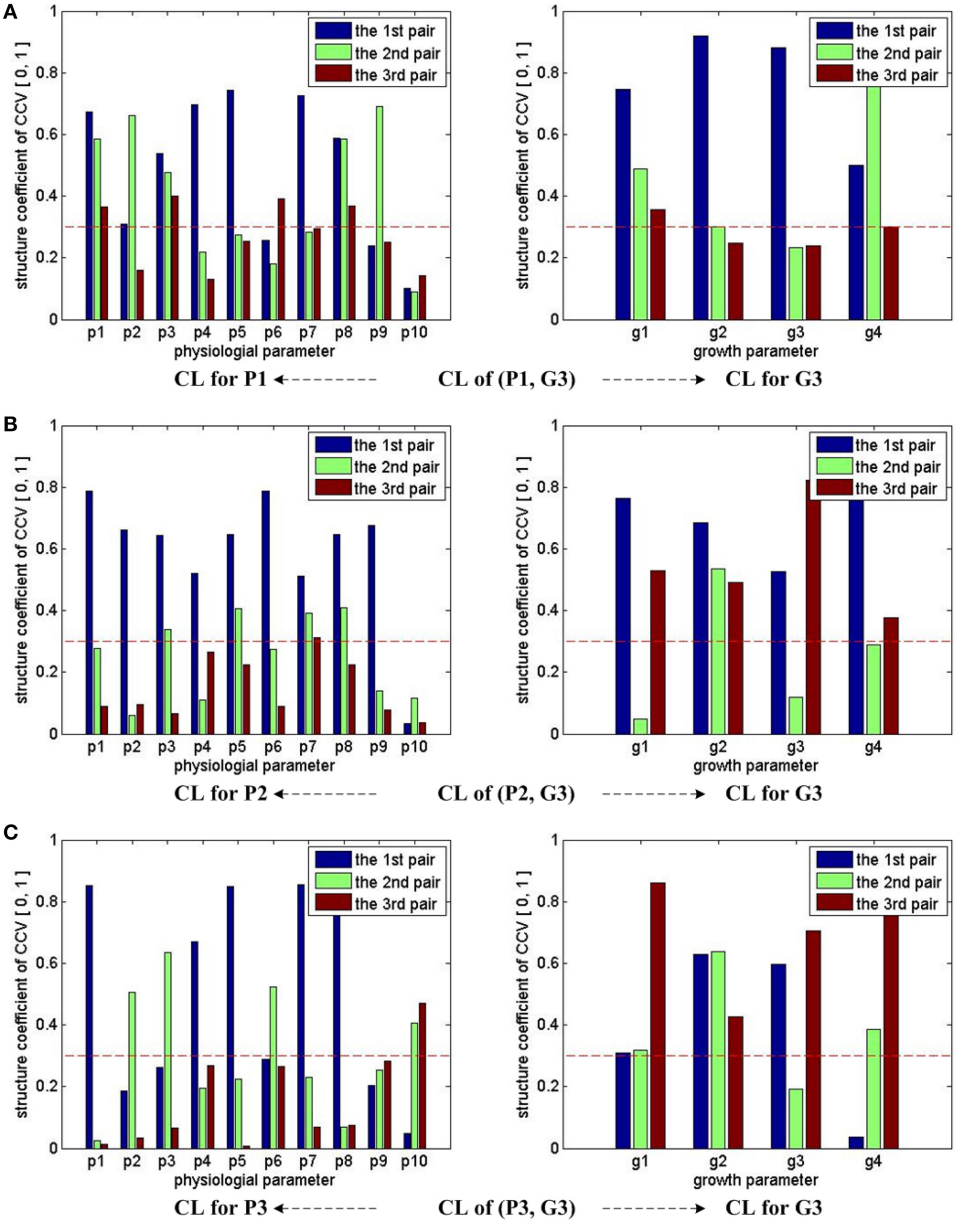
CCA concerned CL bars for (P1, G3), (P2, G3), and (P3, G3). For (P1, G3), (P2, G3), and (P3, G3), as the first three pairs of CCV are concerned by CCA, we have 3 bars for each parameter in all sub-figures. For each parameter, if one bar reaches the red dash line, we conclude that the corresponding parameter has correlations with the other parameter set in the CCA. Taking the sub-figure **(A)** CL of group (P1, G3)—CL for P1 (the sub-figure on the left of the first row) for example, none of p10's bar reaches the dash line, we conclude that the physiological parameter p10 (VpdL) in P1 has no/weak correlation with growth parameters in G3. Following the same rule, we can infer that p10 (VpdL) in P2 and p9 (Tr) in P3 also has no/weak correlation with growth parameters in G3. All growth parameters in G3 have correlation with physiological parameters.

Figure 4 shows the CCA concerned CL of groups (P1, G3), (P2, G3), and (P3, G3). With the help of the marked threshold line, we conclude: for (P1, G3), almost all physiological parameters have influences on growth parameters, except p10; for (P2, G3), almost all physiological parameters have influences on growth parameters, except p10; for (P3, G3), almost all physiological parameters have influences on growth parameters, except p9. In other words, p9 and p10's influences on final growth parameters are not strong enough during the whole experiment period.

Based on the CCA results of the last three (P, G) groups, a new simplified physiological parameter combination without using p9 and p10 is given by

(21) $$\begin{array}{r} \text{P}\_8\text{D}\_\text{9}\_\text{10}=\left[p1,\;p2,\;p3,\;p4,\;p5,\;p6,\;p7,\;p8\right] \end{array}$$

P_8D_9_10 can be used for predicting G3. If P_8D_9_10 delivers similar performances as P_10D, a simplified observer can be built with P_8D_9_10.

### Results of support vector machine prediction

In the control loops of greenhouse-crop systems, observers are used to monitor the states of control objects (environment or crops). If observers find that control objects are not in good states, control strategies will take actions to help objects revert to good states. When the states of objects cannot be monitored directly, prediction observers are used. Here, we use SVM to build crop growth state observers. In the observers, environmental and physiological parameters are inputs and “good” or “bad” states of growth parameters are outputs. The labeling rule for “good” or “bad” growth states is: if one instant of a sample parameter variant is not smaller than the mean value of all instants, it is labeled as “good”; otherwise, “bad.” Using variant Plant_Height1 as an example, we express the labeling rule as

(22) $$\begin{array}{r} \text{sign}\left(g_{i}-\bar{g}\right)=\{\begin{array}{c} 1\;(g_{i}-\bar{g})\geq 0\\ -1\;(g_{i}-\bar{g})<0 \end{array} \end{array}$$

where *g*_*i*_ is the instant of Plant_Height1 in the *i*-th sample, $\bar{g}$ is the mean value of all Plant_Height1 instances. As mentioned in Section Materials and Methods, we have 73 samples of data in total. Each sample has 1 set of environmental parameter variants, 4 sets of physiological parameter variants and 3 sets of growth parameter variants. After labeling, we have 73 classification labels for each growth parameter variant.

To demonstrate the advantages of designing observers based on crop physiological response information, we build three kinds of SVM observers and test them with only environmental parameters, only physiological parameters, and environmental parameters + physiological parameters, respectively. To illustrate the generality of the new observers, we consider three SVM model building strategies, including linear core SVM predictor, RBF core SVM predictor, and RBF core SVM predictor with auto training and cross validation. Furthermore, simplified versions of parameter combinations are tested to illustrate the feasibility of the CCA results. For all the 73 samples, 48 samples are used for SVM model training and 25 samples are used for testing.

#### Testing results for SVM models with RBF core

**Figure 5:**
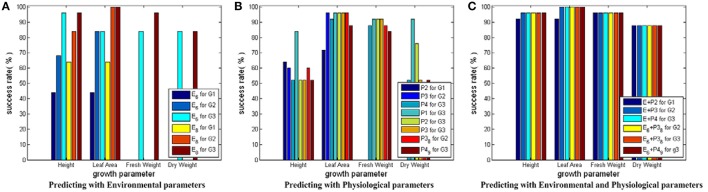
Testing results for SVM modes with RBF core. The height of a bar stands for the prediction success rate of a SVM model. In **(A)** for Height and Leaf_Area, there are 6 bars. The first three bars stand for the success rates of the simplified environmental parameter combinations for predicting the growth parameters in 3 different growth stage—(0~3 days, 4~6 days, and 7~9 days). The last three bars stand for the success rates of the complete environmental parameter combinations for predicting the growth parameters in the final growth stage—(7~9 days). For Fresh_Weight and Dry_Weight, there are 2 bars, one for the success rates of the simplified environmental parameter combination, the other for the success rates of the complete environmental parameter combination. In **(B)** there are 8 bars for each growth parameter. The first three bars stand for the success rates of predicting the growth parameters of different stages with the physiological parameter combinations sampled at the same stage. The fourth to sixth bars stand for the success rates of predicting the growth parameters of the final stage with the physiological parameter combinations sampled at different stages. The last two bars stand for the success rates of predicting the growth parameters of different stages with the simplified physiological parameter combinations sampled at the same stage. In **(C)** there are 6 bars for each growth parameter. The first three bars stand for the success rates of predicting the growth parameters with the complete environmental and physiological parameter combinations. The last three bars stand for the success rates of predicting the growth parameters with the simplified environmental and physiological parameter combinations.

Figure 5 shows the testing results for SVM models with RBF core. When environmental parameters are employed to predict growth parameters, the following 6 SVM models are trained and tested: E_6D (E_6_ in Figure 5A) for predicting G1, E_6D for predicting G2, E_6D for predicting G3, E_8D (E_8_ in Figure 5A) for predicting G1, E_8D for predicting G2, and E_8D for predicting G3. The first 3 models are used to show the performance of the simplified environmental parameter combinations for predicting cucumber seedling growth states in different stages. The last 3 models are used to show the performance of the complete environmental parameter combinations for predicting cucumber seeding growth states in different stages. As each sample only has 1 fresh weight measurement and 1 dry weight measurement, Figure 5A only has 2 bars (E_6_ for predicting G3 and E_8_ for predicting G3) for fresh weight and dry weight, respectively. When physiological parameters are employed to predict growth parameters, the following 8 SVM models are trained and tested: P2 for predicting G1, P3 for predicting G2, P4 for predicting G3, P1 for predicting G3, P2 for predicting G3, P3 for predicting G3, P3_8D_6_9 (P3_8_ in Figure 5B) for predicting G2, and P4_9D_9 (P4_9_ in Figure 5B) for predicting G3. Here, the first 3 models are used to show the performance of physiological parameters for reflecting current cucumber seedling growth states. The fourth to sixth models are used to show performance of physiological parameters in different stages for predicting final cucumber seedling growth states. The last 2 models are used to show the performance of the simplified physiological parameter combinations for reflecting current cucumber seedling growth states. When environmental and physiological parameters are both employed to predict growth parameters, another 6 SVM models are trained and tested: E_8D plus P2 (E+P2 in Figure 5C) for predicting G1, E_8D plus P3 (E+P3 in Figure 5C) for predicting G2, E_8D plus P4 (E+P4 in Figure 5C) for predicting G3, E_6D plus P3_8D_6_9 (E_6_+P3_8_ in Figure 5C) for predicting G2, E_6D plus P3_8D_9_10 (E_6_+P3_8_ in Figure 5C) for predicting G3, and E_6D plus P4_9D_9 (E_6_+P4_9_ in Figure 5C) for predicting G3. Here, the first 3 models are used to show the performances of the complete parameter combinations for predicting cucumber seedling growth states. The last 3 models are used to show the performance of the simplified parameter combinations for predicting cucumber seeding growth states. In the following part for SVM with linear core and SVM with RBF core plus auto training and cross validation, we use the same environmental, physiological and environmental plus physiological parameter combination strategies.

From the prediction success rate of SVM models with RBF core, we conclude that: first, the model performance suffers from dramatic undulation when only environmental parameters are used as the inputs for predicting Height and Leaf_Area, and the prediction accuracy ranges from lower than 50% to higher than 90%; second, the prediction accuracies on Height and Leaf_Area show a rising trend from early stage to late stage of cucumber seedling growth, which implies the influences of environment parameters on crops require time to exhibit their effects; third, the model performance is not good when only physiological parameters are used as the inputs for predicting Height and Dry_Weight, and most model prediction accuracies are around 50%; fourth, the model performance is greatly improved when both environmental and physiological parameters are used as the inputs, and all model prediction accuracies are increased to around 90% and above; fifth, the simplified parameter combinations yield worse performance than the complete parameter combinations unless both the environmental and physiological parameters are used.

#### Testing results for SVM models with linear core

**Figure 6:**
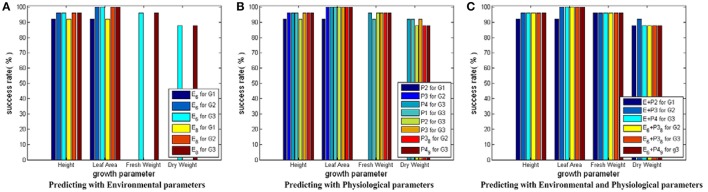
Testing results for SVM modes with linear core. Here, all the SVM models share the same input and output parameter sets as those in Figure 5. Only the core function of the SVM model is changed to linear core. In **(A)** for Height and Leaf_Area, there are 6 bars, respectively. The first three bars show the predicting results using the simplified environmental parameter sets as inputs. The last three bars show the results of the complete environmental parameter sets. For Fresh_Weight and Dry_Weight, there are 2 bars, respectively: one for the simplified environmental parameter combination, the other for the complete environmental parameter combination. In **(B)** there are 8 bars for each growth parameter: the first three for predicting current growth states, the fourth to sixth for predicting the final growth state, and the last two for predicting with the simplified physiological parameter combinations. In **(C)** there are 6 bars for each growth parameter: The first three for predicting with the complete environmental and physiological parameter combinations, the last three for predicting with the simplified environmental and physiological parameter combinations.

Figure 6 shows the testing results for SVM models with linear core. Based on Figure 6, we conclude the following: first, SVM models with linear core can give good and stable prediction performance with success rates around 90% and above; second, the prediction accuracies on Height and Leaf_Area show a rising trend from early stage to late stage of cucumber seedling growth, which implies the influences of environment and physiological parameters on crops require time to exhibit their effects; third, the prediction performance can be slightly improved when physiological parameters are introduced in; fourth, the simplified parameter combinations can bring similar growth estimations compared with the complete parameter combinations, which verifies the feasibility of the CCA results.

#### Testing results for SVM models with RBF core plus auto train and cross validation

**Figure 7:**
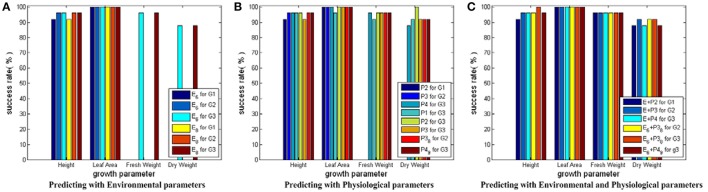
Testing results for SVM modes with RBF core plus auto train and cross validation. Here, all the SVM models share the same input and output parameter sets as those in Figure 5. Only auto train and cross validation techniques are employed during the training process of SVM models with RBF core. In **(A)** for Height and Leaf_Area, there are 6 bars, respectively. The first three bars show the predicting results using the simplified environmental parameter sets as inputs. The last three bars show the results of the complete environmental parameter sets. For Fresh_Weight and Dry_Weight, there are 2 bars, respectively: one for the simplified environmental parameter combination, the other for the complete environmental parameter combination. In **(B)** there are 8 bars for each growth parameter: the first three for predicting current growth states, the fourth to sixth for predicting final growth state, and the last two for predicting with the simplified physiological parameter combinations. In **(C)** there are 6 bars for each growth parameter: The first three for predicting with the complete environmental and physiological parameter combinations, the last three for predicting with the simplified environmental and physiological parameter combinations.

Figure 7 shows the testing results for SVM models with RBF core plus auto train and cross validation. Based on Figure 7, we conclude that: first, this modeling strategy can give even better prediction performance than SVM models with linear core, especially on the prediction for Dry_Weight; second, the physiological parameters show their improving capabilities, which further demonstrate the benefit of incorporating crop physiological responses in the observer building process; third, the feasibility of the CCA results is verified again by the similar performance of the simplified and the complete parameter combinations.

#### Comparison of three different modeling strategies

**Figure 8:**
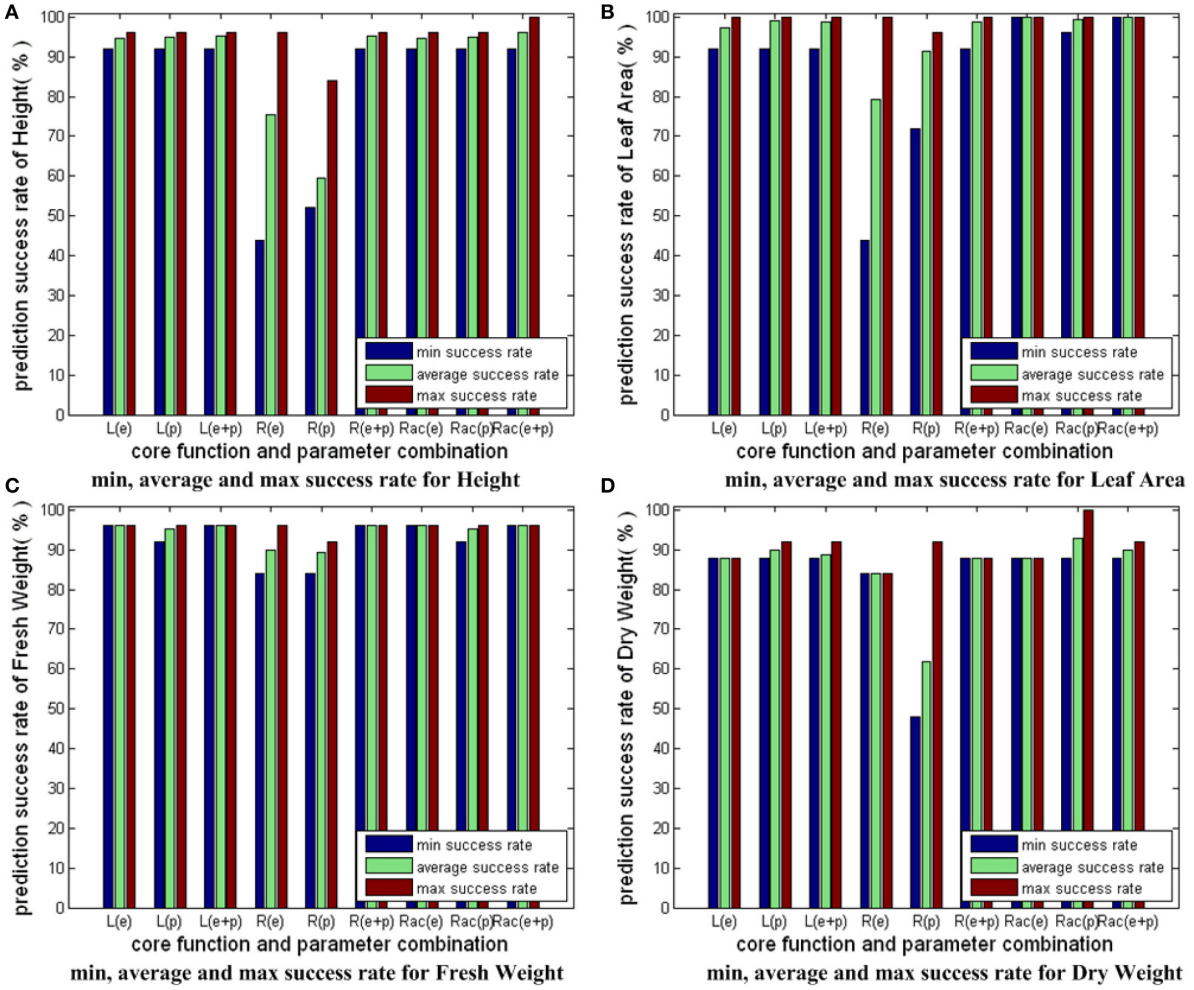
Comparison of three different modeling strategies for 4 growth parameters. The prediction accuracies of different SVM modeling strategies with different input parameter combinations are shown here. We have 3 SVM modeling strategies: SVM with linear core, SVM with RBF core, SVM with RBF core plus auto train and cross validation. Also, we have 3 kinds of input parameter combinations: combinations with only environmental parameters, combinations with only physiological parameters, combinations with both environmental and physiological parameters. As a result, we have 3 × 3 = 9 sets of bars for each sub-figure of growth parameter. For each set, we have 3 bars: the minimum success rate, the average success rate, the maximum success rate. To simplify the expressions, we denote linear core as “L,” RBF core as “R,” RBF core with auto train and cross validation as “Rac,” combinations with only environmental parameters as “e,” combinations with only physiological parameters as “p,” combinations with both environmental and physiological parameters as “e+p.” For example, the bar set marked with “Rac(e+p)” shows the prediction accuracy results of the SVM model with RBF core plus auto train and cross validation, whose input parameters consist of both environmental and physiological parameters.

Figure 8 compares the results in Figures 5–7. For each growth parameter, the SVM models are categorized into 9 groups: 3 SVM models strategies with 3 parameter combination strategies. We denote linear core as “L,” RBF core as “R,” RBF core with auto train and cross validation as “Rac,” parameter combinations consisting of environmental parameters as “e,” parameter combinations consisting of physiological parameters as “p,” parameter combinations consisting of both environmental and physiological parameters as “e+p.” For example, the group marked with “R(e+p)” indicates the prediction results of the SVM models with RBF core that employs both environmental and physiological parameters as inputs. For every group, we compute and show 3 bars: the minimum success rate, the average success rate and the maximum success rate of the group. From Figure 8, we conclude the following: first, “L” and “Rac” are capable modeling strategies for our applications, while “R” is not good enough because the minimum success rate greatly differs from the maximum success rate, and the average success rate is not high; second, the overall performance of “Rac” in the experiment is slightly better than those of “L”; third, models containing physiological parameters usually deliver better performance than those containing only environmental parameters.

**Figure 9:**
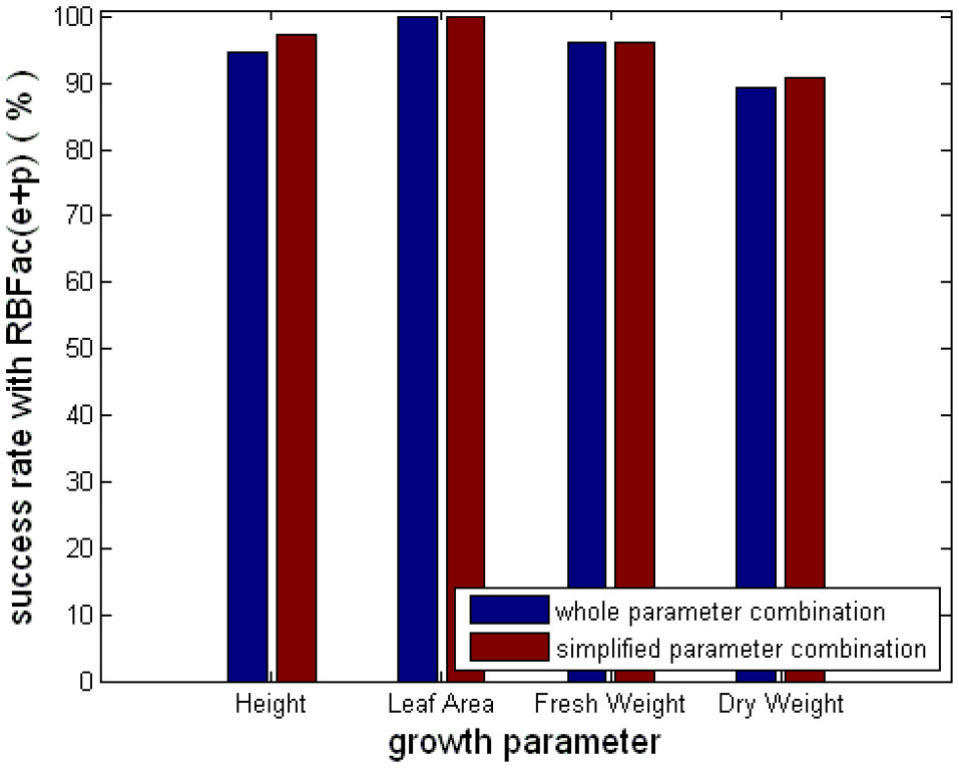
Comparison of the simplified and the complete parameter combinations for the group of “Rac(e+p).”

Figure 9 shows the average prediction success rates of the simplified parameter combinations and the complete parameter combinations for the group of “Rac(e+p).” From Figure 9, we see that the average prediction accuracies of the simplified and the complete parameter combinations are equal for Leaf_Area and Fresh_Weight. The average prediction accuracies of the simplified parameter combinations are better than the complete parameter combinations for Height and Dry_Weight. The results in Figure 9 clearly elaborate that using CCA results, we can simplify parameter combinations for observer models to improve the computational efficiency and feasibility, without reducing observer accuracy.

## Discussions

In this study, we have proposed a new state observer modeling strategy for greenhouse cucumber seedling growth. Our strategy integrates crop physiological information to the modeling process. Using physiological information to improve the inner greenhouse microclimate control is not a new idea. A similar concept named with “Speaking Plant” was presented in 1978 (Udink ten Cate et al., 1978), hoping that control systems could act according to plants' actual requirements. However, due to the limitation in sensing technologies, “Speaking Plant” mainly focused on obtaining physiological information through mathematical derivations. Van Pee and Berckmans built a mathematical model to describe the relationships between physiological parameters and environmental parameters, and employed the model to support the online greenhouse microclimate control (Van Pee and Berckmans, 1998). The physiological parameters in the model were photosynthesis and water potential, and the environmental parameters in the model were CO_2_ concentration and lighting intensity. González-Real and Baille tried to simulate feedbacks of a greenhouse rose crop in the greenhouse control loop, by combining a physiological sub-model together with a physical sub-model. The physiological sub-model contained 3 physiological parameters, including net CO_2_ assimilation, stomatal conductance and transpiration (González-Real and Baille, 2001). As mathematical models stand on the basis of mass/energy transfer equations, they have innate drawbacks on elaborating plants' responses with high accuracy and frequency. By using chlorophyll fluorescence analysis as the physiological sensing tool, our new strategy can give growth state prediction accuracies as high as 90% (Figures 6, 7).

With the development of sensing technologies, chlorophyll fluorescence analysis was developed to monitor the health condition of greenhouse tomato seedlings. It has been reported that visible symptom of physiological dysfunction can detected in the early stage (Takayama et al., 2011a). Also, unhealthy seedlings can be found in the early stage under drought stress (Takayama et al., 2011b). Induction curve, which is generated with the chlorophyll fluorescence intensity changing over time, is the key for dysfunction detection. In our strategy, we take 10 physiological parameters under consideration, including net photosynthesis rate (Pn), stomatal conductance (Cond), intercellular CO_2_ concentration (Ci), efficiency of excitation capture by open PSII center (Fv′/Fm′), quantum efficiency of PSII (PhiPS2), quantum efficiency of CO_2_ fixation (PhiCO_2_), photochemical quenching coefficient (qP), electron transport rate (ETR), transpiration rate (Tr), and vapor pressure deficit at the leaf temperature (VpdL). We believe that more parameters lead to a more comprehensive view of crop responses.

Recently, machine learning technologies appear in the modeling of crop physiological responses. Moriyuki and Fukuda employed neural network to predict the growth state of lettuce seedlings. They took chlorophyll fluorescent intensity, leaf area and circadian rhythms as the inputs for the neural network, and average fresh weight as the output (Moriyuki and Fukuda, 2016). However, as the prediction accuracy was not reported, we cannot compare directly with the neural network model. The prediction accuracy of a neural network strongly depends on the size of the training set. Typically, a satisfying accuracy requires a large training set. Compared with neural network, SVM has distinguishing advantages on handling small sample size problems, nonlinear classification/prediction problems and high dimensional classification/prediction problems. It takes both empirical risk and confidence risk under consideration, compromises between model complexity (learning accuracy) and learning capability (the capability of handling noise, outliers, etc.), and tries to carry out structural risk minimization for classification/prediction tasks. SVM in the current strategy does not need a large training set, which makes our strategy feasible for application. Also, our strategy takes 4 growth parameters into consideration, and feeds more information back to the controller than the previous work (Moriyuki and Fukuda, 2016).

**Figure 10:**
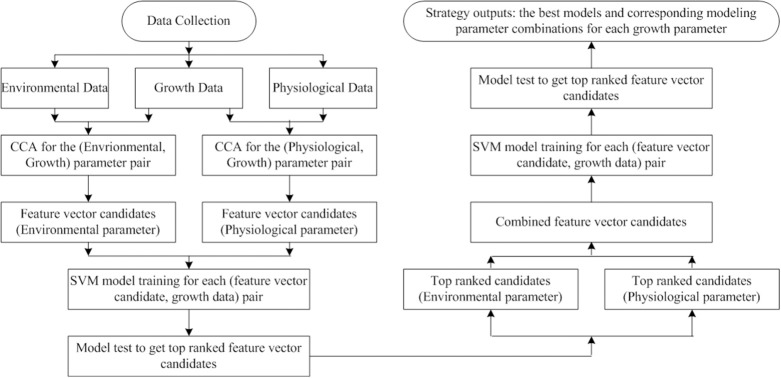
Flow chart for the observer modeling process. Even though we have quite good results using CCA and SVM, we believe that the strategy can be further improved if we use Kernel CCA (KCCA) and SVM. This is because CCA is limited by its linear natural instincts whereas KCCA (Akahu, 2001) is capable for analyzing data coming from nonlinear systems.

Although we use cucumber seedlings as an instance, the proposed observer modeling strategy can be applied to other seedlings as a universal strategy. The strategy consists of 4 steps: (1) Carrying out the data acquisition tasks following the instructions in Section Data Acquisition. Note that, the setting of environmental parameters and sub-experiments should be altered according to practical situations; (2) Canonical Correlation Analysis is launched to reveal the correlations of (environmental, growth) and (physiological, growth) parameter pairs. Based on the CCA results, several parameter combinations are chosen as feature vector candidates for building SVM models. In this step, all the feature vectors are formed by parameters coming from the same category: environmental or physiological; (3) An SVM model is trained for each (feature vector, growth parameter) pair. If there are *m* feature vectors and *n* growth parameters, we will have *m* × *n* SVM models. The modeling strategy with RBF kernel plus auto train and cross validation will be used for all SVM models. The testing results will illustrate the top ranked combinations for environmental and physiological parameters, respectively; (4) Several combined feature vectors are formed to obtain new SVM models. A combined feature vector contains a top ranked environmental combination and a top ranked physiological combination. If there are *r* combined feature vectors, we will have *r* × *n* new SVM models. For each growth parameter, we have *m* + *r* SVM models. Among them, the one with the best testing performance will be chosen as the final model. The flow chart for the whole observer modeling process is shown in Figure 10.

After the observer is obtained, we can add it together with a chlorophyll fluorescent sensor into the greenhouse microclimate control loop.

## Author contributions

KS designed the data collection experiments; CZ and WW carried out the data collection experiments; QQ designed the modeling strategy; QQ and XQ developed the modeling strategy experiment codes; QQ and HB analyzed the modeling strategy experiment results; JY discussed the data and revised the article; QQ, KS, and HB wrote the manuscript.

### Conflict of interest statement

The authors declare that the research was conducted in the absence of any commercial or financial relationships that could be construed as a potential conflict of interest.
